# A viral codon usage strategy enhances antigen production and protection in SFTSV mRNA vaccination

**DOI:** 10.1038/s41541-025-01298-4

**Published:** 2025-11-24

**Authors:** Inho Cha, Yumiko Yamada, Dokyun Kim, Soowon Kang, Wan-Shan Yang, Woo-Jin Shin, Anna Ryder, Morgan Lewis, Chloe Chung, Nam-Hyuk Cho, Young Ki Choi, Jianrong Li, Chih-Jen Lai, Jae U. Jung

**Affiliations:** 1 Department of Infection Biology, Cleveland Clinic, Cleveland, OH USA; 2 Global Center for Pathogen and Human Health Research, Cleveland Clinic, Cleveland, OH USA; 3 Department of Molecular Biology and Microbiology, Case Western Reserve University School of Medicine, Cleveland, OH USA; 4 Department of Microbiology and Immunology, Seoul National University College of Medicine, Seoul, Republic of Korea; 5 Department of Biomedical Sciences, Seoul National University College of Medicine, Seoul, Republic of Korea; 6 Center for Study of Emerging and Re-emerging Viruses, Korea Virus Research Institute, Institute for Basic Science, Daejeon, Republic of Korea; 7 Department of Veterinary Biosciences, College of Veterinary Medicine, Infectious Disease Institute, The Ohio State University, Columbus, OH USA; 8 Institute of BioPharmaceutical Sciences, National Sun Yat-sen University, Kaohsiung, Taiwan, ROC

**Keywords:** RNA vaccines, Virology

## Abstract

Severe Fever with Thrombocytopenia Syndrome Virus (SFTSV) is an emerging tick-borne pathogen with a high case-fatality rate and no approved vaccine, posing a global health threat. Human codon-optimized viral antigens can enhance mRNA vaccine efficacy by improving antigen expression but often requires high mRNA doses, increasing the risk of side effects. In this study, we introduce a codon optimization strategy using Herpes Simplex Virus 1 glycoprotein B (HSVgB) codon usage to develop an mRNA lipid nanoparticle (LNP) vaccine targeting the neutralizing Gn-H domain of SFTSV (sGn-H). The HSVgB codon-optimized mRNA vaccine [sGn-H (HSVgB)] achieved higher antigen expression and elicited stronger humoral and cellular immune responses than a human codon-optimized counterpart [sGn-H (human)]. Notably, sGn-H (HSVgB) induced more bone marrow-resident antibody-secreting cells and conferred superior protection against SFTSV at lower doses. These findings highlight HSVgB codon optimization may represent a promising strategy for enhancing the immunogenicity with low-dose mRNA immunization.

## Introduction

Severe fever with thrombocytopenia syndrome virus (SFTSV), recently renamed as *Bandavirus dabieense*, is a newly emerging tick-borne virus first identified in China in 2009. Since then, SFTSV has spread across East Asia, including Korea, Japan, and Taiwan, and more recently, Vietnam, Myanmar, and Pakistan^1–6^. While human-to-human and animal-to-human transmission via blood exposure have been reported, the primary mode of infection remains tick bites, particularly from *Haemaphysalis* (*H.) longicornis*^7–10^. Initial symptoms of SFTSV infection include fever, myalgia, and fatigue, which can progress to severe symptoms like hemorrhage, thrombocytopenia, and leukopenia^11,12^. The case fatality rate ranges from 10 to 30%, with older adults (≥50 years) being at higher risk^7,11,13,14^. Due to its high mortality and the geographic expansion of *H. longicornis* into regions beyond Asia, such as Australia, New Zealand, and the United States, SFTSV is listed as a priority pathogen by both the United States National Institute of Allergy and Infectious Diseases and the World Health Organization^15,16^. Unfortunately, no FDA-approved vaccines for SFTSV are currently available.

SFTSV, a member of the genus *Bandavirus* in the family *Phenuiviridae* (order *Hareavirales*), has a tripartite, single-stranded, negative-sense RNA genome composed of large (L), medium (M), and small (S) segments^11^. Among these, the M segment has been considered an attractive target for vaccine development due to its role in viral entry and immunogenicity^17–21^. The M segment encodes the viral glycoproteins, Gn and Gc, which form heterodimers and assemble into an icosahedral shell on the virion membrane^22,23^. The Gn protein also facilitates host cell attachment by binding to the C-C motif chemokine receptor 2 (CCR2)^24^. Moreover, neutralizing antibodies from SFTSV convalescent patients have been mapped to the Gn head (Gn-H) domain^25,26^. Our previous studies demonstrated that SFTSV Gn-H (sGn-H), using ferritin nanoparticles and mRNA vaccine platforms, protects against lethal SFTSV challenge, supporting Gn-H as a promising vaccine antigen^21,27^.

mRNA vaccines gained prominence during the COVID-19 pandemic due to advantages such as flexibility in antigen design, ease of production, and efficient antigen expression through host translational machinery^28,29^. These features make mRNA platforms compelling for SFTSV vaccine development. Previous studies have investigated mRNA-based vaccine candidates against SFTSV using doses ranging from 1 to 10 µg, with 5 and 10 µg doses notably eliciting strong immune responses and protection^19,20^. However, higher mRNA doses may be associated with potential adverse effects, probably due to immune overstimulation triggered by exogenous mRNAs and/or lipid nanoparticle components^30^. In fact, phase I and II clinical trials of Pfizer’s and Moderna’s SARS-CoV-2 mRNA vaccines reported a wide range of adverse effects, with more severe reactions observed at higher doses^31,32^. Therefore, optimizing the vaccine dose to the minimal level that elicits robust immunogenicity is crucial for reducing the risk of adverse effects in future mRNA vaccine development.

One strategy to improve mRNA vaccine performance is codon optimization, which involves modifying viral gene codons to match those preferentially used by the host. Codon preferences differ among species due to variations in cognate tRNA availability or tRNA gene copy number^33,34^. Thus, aligning codon usage to match host preferences can enhance translation efficiency and protein expression^35–38^. However, excessive optimization toward the most frequent codons can accelerate translation to the point where co-translational folding is impaired, which can result in misfolded proteins that are targeted for degradation, reducing the amount of functional antigen and diminishing immune responses^34,39–42^.

To avoid this problem, we explored a balanced codon usage strategy that maintains efficient translation while retaining rare codons to preserve protein integrity. We hypothesized that the codon usage of Herpes simplex virus 1 (HSV-1) glycoprotein B (HSVgB) could serve this purpose. HSVgB is highly expressed during the late stage of infection, when host translation machinery is under viral control, and its codon usage naturally contains a mixture of frequent and rare codons. This pattern likely reflects an evolutionary adaptation to achieve high protein expression while maintaining folding integrity^43–45^. Based on this rationale, our previous study showed that a SARS-CoV-2 Spike mRNA vaccine optimized with HSVgB codons induced markedly high antigen expression and stability, along with enhanced total IgG, neutralizing antibody, and T cell responses, compared to a vaccine optimized with human codons, ultimately providing improved protection against lethal SARS-CoV-2 challenge^46^. These findings support the potential of a novel, persistent herpesviral codon-usage platform for mRNA vaccines aimed at improving antigen expression and long-term protection against lethal viral infections.

In this study, we applied either human or HSVgB codon usage to optimize the sGn-H gene and compared their vaccine performance using a low dose (1 µg) of mRNA-LNPs. The sGn-H (HSVgB) mRNA demonstrated significantly higher antigen expression than sGn-H (human) mRNA and elicited stronger humoral and cellular immune responses in mice. Furthermore, sGn-H (HSVgB) vaccination conferred superior protection against SFTSV challenge compared to the human codon-optimized version. These results highlight HSVgB codon optimization as a promising approach to enhance mRNA vaccine efficacy and protective immunity.

## Results

### Design and characterization of the sGn-H mRNA vaccines

**Fig. 1:**
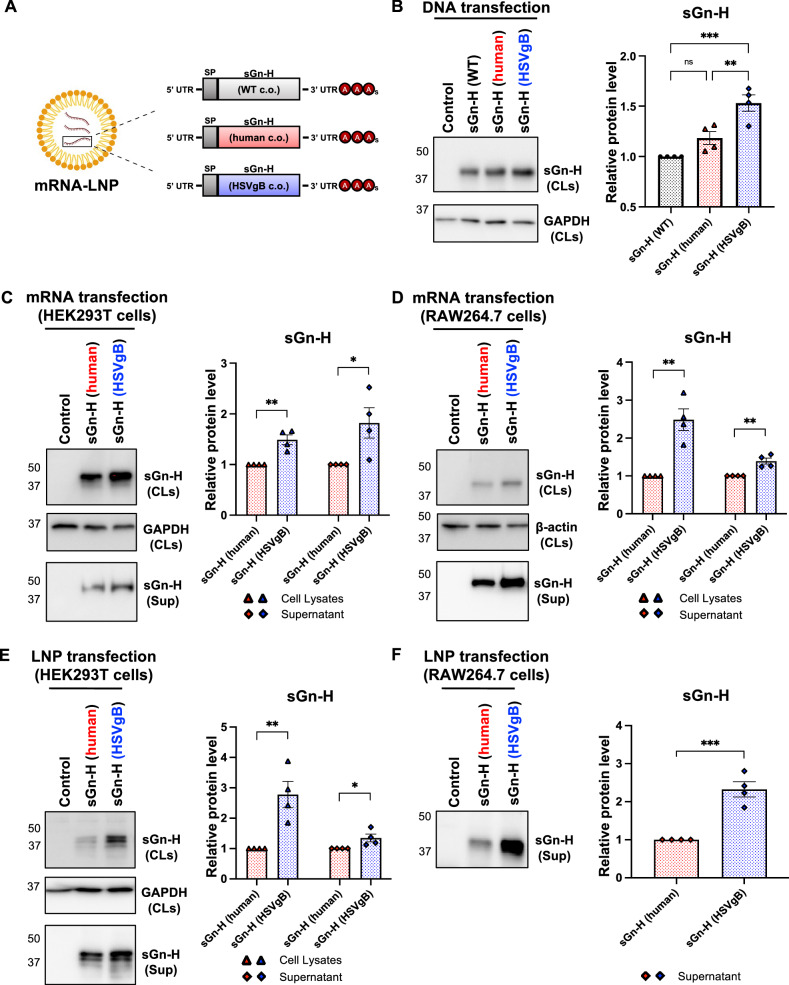
Design of the SFTSV Gn-H mRNA vaccines. **A** Schematic representation of mRNA vaccines encoding sGn-H. The sGn-H sequences were codon-optimized for the original wild-type sequence, human codon usage, or HSVgB codon usage. These genes were flanked by a 5’ untranslated region (UTR), a signal peptide (SP), a 3’ UTR, and poly(A) tails. The sGn-H mRNAs were encapsulated in lipid nanoparticles (LNPs). “C.o.” stands for codon-optimized. **B** Western blot analysis of sGn-H expression in cell lysates following DNA transfection. The “Control” lane represents mock transfection. “sGn-H (WT)” refers to the original WT sGn-H sequence, “sGn-H (human)” to the human codon-optimized sGn-H antigen, and “sGn-H (HSVgB)” to the HSVgB codon-optimized sGn-H antigen. Protein expression levels were normalized to GAPDH, and relative protein expression was compared to sGn-H (WT). **C**, **D** Western blot analysis of sGn-H expression in cell lysates and supernatants following mRNA transfection in HEK293T and RAW264.7 cells. Protein levels in HEK293T cell lysates were normalized to GAPDH, and RAW264.7 cell lysates to β-actin. Relative expressions were compared to sGn-H (human). “sGn-H (CLs)” refers to sGn-H in cell lysates. “GAPDH (CLs)” to GAPDH from HEK293T cell lysates, “β-actin (CLs)” to β-actin from RAW264.7 cell lysates, and “sGn-H (Sup)” to sGn-H in supernatants. **E**, **F** Western blot analysis of sGn-H expression in cell lysates and supernatants following LNP transfection. Protein levels in HEK293T cell lysates were normalized to GAPDH. The relative protein expression was compared to sGn-H (human). Protein band intensities were quantified using ImageJ software (NIH). Statistical analysis was determined by one-way ANOVA with Šídák’s post hoc multiple comparison test for DNA transfection, and by Student’s t-test for mRNA and LNP transfection. *p* ≤ 0.05 is denoted by *, p ≤ 0.01 by **, p ≤ 0.001 by ***, and ns indicates no significant difference. **A** image was created with BioRender.com.

We designed our SFTSV mRNA vaccines using the neutralizing Gn-H domain of the SFTSV Gn glycoprotein (Fig. 1A). Each sGn-H construct was codon-optimized to the viral wild-type sequence [sGn-H (WT)], human codon usage [sGn-H (human)], or HSVgB codon usage [sGn-H (HSVgB)]. The full sequences, GC content, and dinucleotide motif frequencies of sGn-H (human) and sGn-H (HSVgB) are provided in Supplementary Tables 1 and 2. These codon-optimized sGn-H genes were cloned into expression plasmids containing mRNA components as previously described^21^. To evaluate protein expression, plasmids encoding each sGn-H construct were transfected into HEK293T cells. Western blot analysis showed that sGn-H (HSVgB) had the highest protein accumulation in cells, followed by sGn-H (human), with the lowest expression observed in sGn-H (WT) (Fig. 1B). Based on these results, we selected sGn-H (HSVgB) and sGn-H (human) as mRNA vaccine candidates for further evaluation.

sGn-H mRNAs were synthesized via in vitro transcription (IVT) using T7 RNA polymerase, and poly(A) tails were enzymatically added to the 3’ ends. The synthesized sGn-H (human) and sGn-H (HSVgB) mRNAs were then transfected into HEK293T and RAW264.7 cells, and both cell lysates and supernatants were collected to measure intracellular accumulation and secretion, respectively. Western blot analyses revealed that sGn-H (HSVgB) mRNAs led to greater sGn-H accumulation in cell lysates and higher levels in supernatants than sGn-H (human) mRNAs (Fig. 1C, D). A similar trend was observed with sGn-H mRNA-lipid nanoparticle (mRNA-LNP) transfection, exhibiting 4-fold increase in total sGn-H expression from HEK293T cells and 2.3-fold increase in secreted sGn-H levels in the supernatant from RAW264.7 cells (Fig. 1E, F). These results indicate that sGn-H (HSVgB) antigen design yields higher protein accumulation and secretion than the sGn-H (human) design.

### Elevated antibody responses in mice immunized sGn-H (HSVgB) mRNA-LNP vaccination

**Fig. 2:**
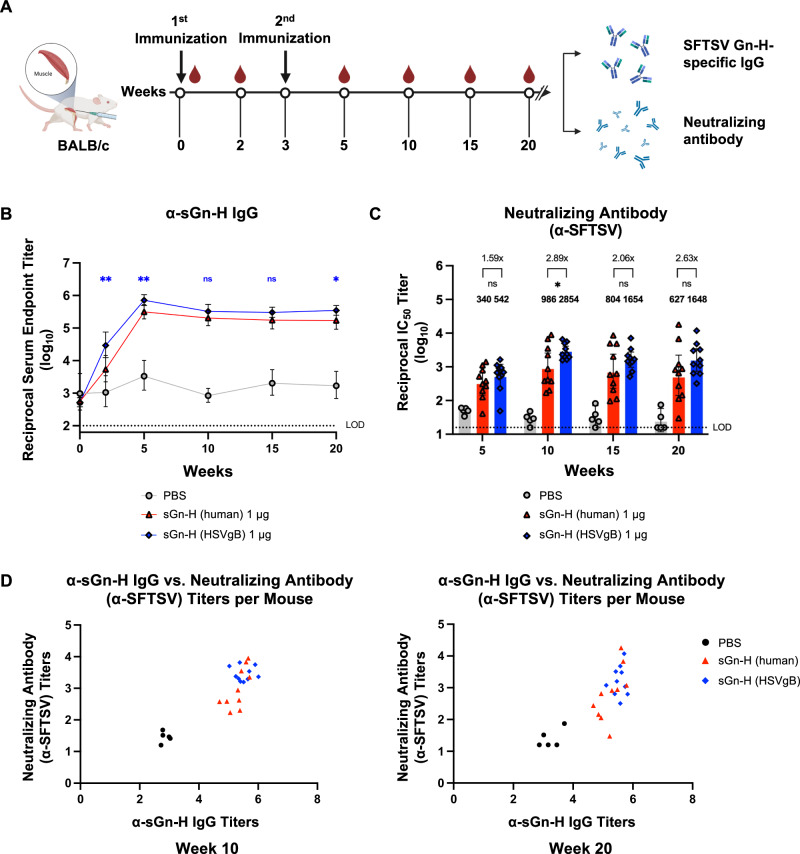
BALB/c mice immunized with sGn-H (HSVgB) mRNA-LNPs produced elevated levels of sGn-H-specific IgG and neutralizing antibodies. **A** Experimental design and timeline of BALB/c mice immunization. Three groups of 6–8-week-old female BALB/c mice were immunized intramuscularly with PBS (*n* = 5), 1 μg of sGn-H (human) mRNA-LNP (*n* = 10), or 1 μg of sGn-H (HSVgB) mRNA-LNP (*n* = 10) at weeks 0 and 3. Sera were collected at weeks 0, 2, 5, 10, 15, and 20 for antibody titers measurement. **B** Endpoint enzyme-linked immunosorbent assay (ELISA) was used to measure the reciprocal serum endpoint titer of total IgG antibody recognizing sGn-H. Statistical analysis was performed comparing sGn-H (human) mRNA-LNP with sGn-H (HSVgB) mRNA-LNP. The dotted line represents the limit of detection (LOD). **C** A pseudovirus neutralization assay was used to measure the reciprocal IC_50_ titer of neutralizing antibodies against a recombinant vesicular stomatitis virus (rVSV) pseudovirus expressing the HB29 strain of SFTSV glycoprotein (SFTSV G) and luciferase reporter gene. The fold difference between sGn-H (human) mRNA-LNP and sGn-H (HSVgB) mRNA-LNP is written above the statistical significance. The geometric mean titers are shown below the statistical difference in bold. Statistical analysis was performed comparing sGn-H (human) mRNA-LNP with sGn-H (HSVgB) mRNA-LNP. The dotted line represents LOD. **D** Comparison of total IgG antibodies recognizing sGn-H and neutralizing antibodies was made for individual mice at weeks 10 and 20. Statistical analysis was determined by Student’s t-test for total IgG antibody recognizing sGn-H and neutralization antibodies. *p* ≤ 0.05 is denoted by *, *p* ≤ 0.01 by **, and *ns* indicates no significant difference. **A** image was created with BioRender.com.

Antibodies protect against pathogens by neutralizing them or participating in complement-dependent cytotoxicity and antibody-dependent phagocytosis^47^. To compare the efficacy of HSVgB codon-optimized versus human codon-optimized mRNA vaccines, we measured antibody titers following immunization in mice. Female BALB/c mice were vaccinated intramuscularly with PBS (control), 1 µg of sGn-H (human) mRNA-LNP, or sGn-H (HSVgB) mRNA-LNP at weeks 0 and 3 (Fig. 2A). Sera were collected at weeks 0, 2, 5, 10, 15, and 20 to evaluate sGn-H-specific IgG and neutralizing antibody titers against SFTSV, thereby assessing both the short-term response (post-primary and booster immunization) and long-term durability of the antibody response. Compared to sGn-H (human) mRNA-LNP immunized mice, sGn-H (HSVgB) mRNA-LNP immunized mice generated significantly higher total anti-sGn-H IgG levels after both the primary (week 2) and booster (week 5) immunizations (Fig. 2B). While both vaccinated groups maintained anti-sGn-H antibody titers through week 20, sGn-H (HSVgB) consistently produced higher levels than sGn-H (human).

To assess neutralizing activity, we performed a pseudovirus neutralization assay using recombinant VSV pseudovirus expressing SFTSV Gn/Gc glycoproteins and a luciferase reporter gene (rVSV-SFTSV G-Luc)^21,27^. Consistent with total anti-sGn-H IgG results, sGn-H (HSVgB) mRNA-LNP immunization elicited higher geometric mean neutralizing antibody titers than sGn-H (human) mRNA-LNP, with a 2.06- to 2.89-fold difference observed between weeks 10 and 20 (Fig. 2C). Analysis of antibody production in individual mice further confirmed that sGn-H (HSVgB) mRNA-LNP induced stronger and more consistent titers of both total anti-sGn-H IgG and neutralizing antibodies at weeks 10 and 20 compared to sGn-H (human) mRNA-LNP (Fig. 2D). Collectively, these results demonstrate that sGn-H (HSVgB) mRNA-LNP vaccination elicits stronger and more durable antibody responses than sGn-H (human) mRNA-LNP.

### Enhanced bone marrow antibody responses in sGn-H (HSVgB) mRNA-LNP immunized mice

**Fig. 3:**
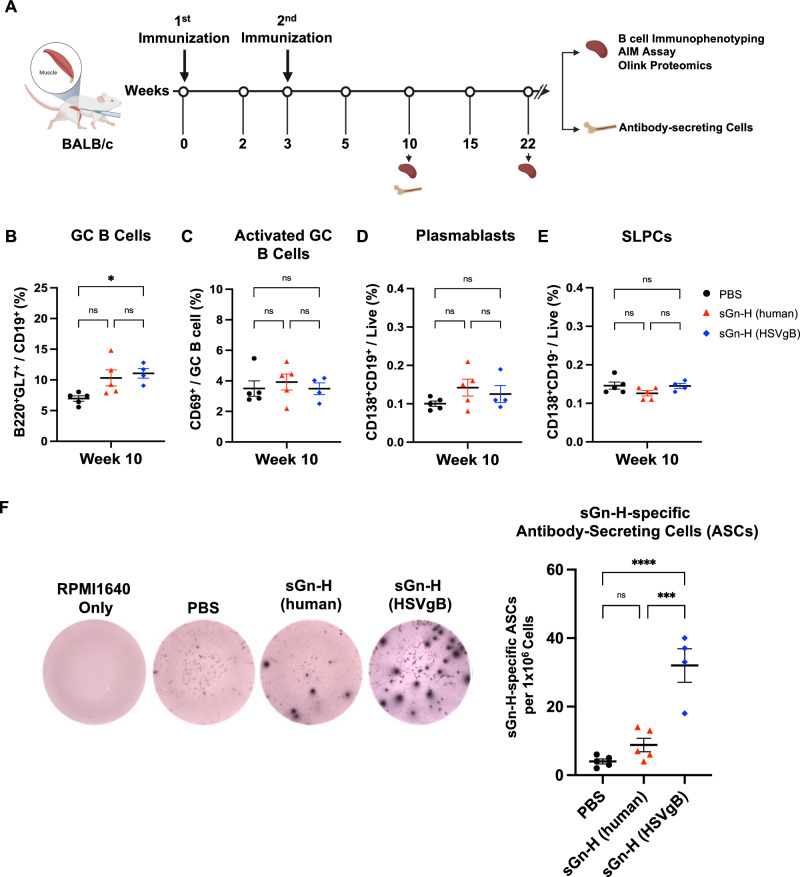
sGn-H (HSVgB) mRNA-LNP-immunized BALB/c mice show similar spleen B cell populations but increased sGn-H-specific antibody-secreting cells (ASCs) in the bone marrow. **A** Experimental design and timeline of BALB/c mice immunization. Three groups of 6–8-week-old female BALB/c mice (*n* = 5 per group) were immunized intramuscularly with PBS, 1 μg of sGn-H (human) mRNA-LNP, or 1 μg of sGn-H (HSVgB) mRNA-LNP at weeks 0 and 3. Splenocytes were harvested at weeks 10 and 22, and bone marrow cells were harvested at week 10. One mouse from the sGn-H (HSVgB) mRNA-LNP group at week 10 was excluded as an outlier and not included in the analyses. Splenocytes collected at week 10 were analyzed by flow cytometry to determine the percentages of **B** germinal center (GC) B cells, **C** activated GC B cells, **D** plasmablasts, and **E** short-lived plasma cells (SLPCs). **F** Enzyme-linked immunospot (ELISpot) assay was used to quantify sGn-H-specific ASCs in the mouse bone marrow at week 10. Representative images of ELISpot wells from each mouse group are shown. Statistical analysis was determined by one-way ANOVA with Šídák’s post hoc multiple comparison test for B cell immunophenotyping and ELISpot assay. *p* ≤ 0.05 is denoted by *, *p* ≤ 0.001 is denoted by ***, *p* ≤ 0.0001 by ****, and *ns* indicates no significant difference. **A** image was created with BioRender.com.

Following viral infection or immunization, B cells can recognize antigens, differentiate into plasma cells to produce antibodies, or become memory B cells to mediate long-term immunity^48,49^. Given the robust antibody responses observed following sGn-H mRNA-LNP vaccination, we next sought to characterize the B cell subsets involved in this response. Splenocytes were harvested at week 10 post-primary immunization, and B cell immunophenotyping was performed via flow cytometry (Fig. 3A). The gating strategy for B cell populations is shown in Fig. S1A. We first examined transitional type 1 (T1), type 2 (T2), and mature B cell populations, which represent B cell maturation stages in the spleen^49,50^. At week 10, there were no difference in T1 and T2 B cell populations among the groups, while mature B cell populations were slightly higher in PBS-immunized mice (Fig. S1B–D). We next investigated germinal center (GC) B cells and activated GC B cells, which are critical for enhancing antibody affinity by somatic hypermutation and class switching and ultimately transitioning into plasma and memory B cells^49,51^. Although sGn-H (HSVgB) mRNA-LNP mice had more GC B cell populations than PBS-immunized mice, no statistically significant differences were observed between sGn-H (human) and sGn-H (HSVgB) mRNA-LNP groups (Fig. 3B). In addition, there was no statistical difference in activated GC B cell populations among the three immunized groups at week 10 (Fig. 3C).

Lastly, we examined plasmablasts and short-lived plasma cells (SLPCs), which are antibody-secreting cells (ASCs) generated during viral infection or vaccination^52^. At week 10, no significant differences in plasmablasts or SLPC populations were observed between the two immunized groups or compared to the control (Fig. 3D, E).

SLPCs are transient and predominantly reside in the spleen, whereas long-lived plasma cells (LLPCs) migrate to the bone marrow and sustain long-term antibody production^49,53,54^. Therefore, despite no observed differences in splenic plasma cell subsets, we evaluated ASC responses in the bone marrow, which is a more relevant indicator of durable humoral immunity. At week 10, sGn-H-specific ASCs in the bone marrow were quantified using ELISpot (Fig. S2A). Strikingly, the number of spot-forming units of sGn-H-specific ASCs was 3.5-fold higher in the bone marrow cells of sGn-H (HSVgB) mRNA-LNP immunized mice compared to those of sGn-H (human) mRNA-LNP immunized group (Fig. 3F). These results suggest that although splenic B cell populations were largely similar across the vaccinated groups, sGn-H (HSVgB) mRNA-LNP immunization led to enhanced sGn-H-specific antibody production by bone marrow-resident ASCs, which are critical for long-term humoral immunity.

### sGn-H (HSVgB) mRNA-LNP induces robust CD4^+^ and CD8^+^ T cell responses in mice

**Fig. 4:**
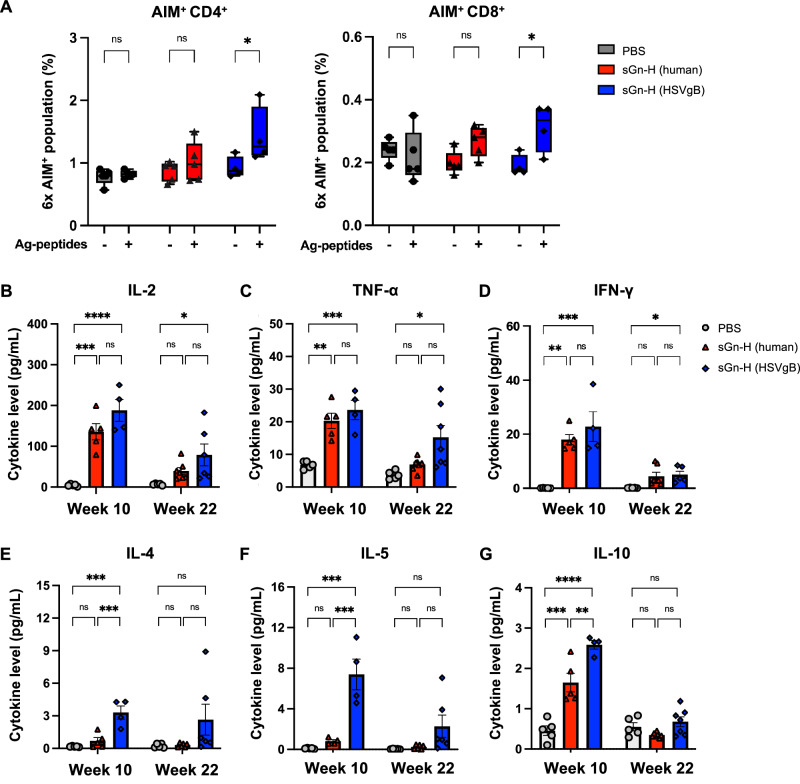
sGn-H (HSVgB) mRNA-LNPs induce stronger T cell immune responses in BALB/c mice. **A** A 6X multiplexed T cell activation-induced marker (AIM) assay was used to identify sGn-H-specific CD4^+^ and CD8^+^ T cells. Splenocytes from BALB/c mice (*n* = 5 per group) immunized with PBS, sGn-H (human) mRNA-LNP, or sGn-H (HSVgB) mRNA-LNP were harvested at week 10. These splenocytes were stimulated with sGn-H overlapping peptide pools to activate sGn-H-specific CD4^+^ and CD8^+^ T cells, and the T cell populations were compared with unstimulated splenocytes. One mouse from sGn-H (HSVgB) mRNA-LNP group was excluded as an outlier. “Ag-peptides –” indicates no peptide stimulation, while “Ag-peptides +” indicates stimulation with overlapping peptide pool. Olink Proteomics were used to measure cytokine concentrations. Splenocytes from BALB/c mice immunized with PBS (*n* = 5), sGn-H (human) mRNA-LNP (*n* = 5), or sGn-H (HSVgB) mRNA-LNP (*n* = 5) were harvested at week 10, and from PBS (*n* = 5), sGn-H (human) mRNA-LNP (*n* = 7), or sGn-H (HSVgB) mRNA-LNP (*n* = 7) at week 22. These splenocytes were stimulated with sGn-H overlapping peptide pools, and the supernatants were collected for analysis of Th1 cytokines, including **B** IL-2, **C** TNF-α, and **D** IFN-γ, and Th2 cytokines, including **E** IL-4, **F** IL-5, and **G** IL-10. One mouse from sGn-H (HSVgB) mRNA-LNP group was excluded as an outlier at week 10. Outliers in B) to G) were calculated in GraphPad Prism v10 and excluded. Statistical analysis was determined by two-way ANOVA with Šídák’s post hoc multiple comparison test for T cell AIM assay, and by one-way ANOVA with Šídák’s post hoc multiple comparison test for Olink proteomics. *p* ≤ 0.05 is denoted by *, *p* ≤ 0.01 by **, *p* ≤ 0.001 by ***, *p* ≤ 0.0001 by ****, and *ns* indicates no significant difference.

To evaluate the T cell response induced by sGn-H mRNA vaccines, we performed a multiplex T cell activation-induced marker (AIM) assay to detect sGn-H-specific CD4^+^ and CD8^+^ T cells. Splenocytes harvested at week 10 post-primary immunization were stimulated ex vivo with an overlapping peptide pool spanning the sGn-H sequence and analyzed by flow cytometry. The gating strategy for identifying AIM^+^ CD4^+^ and CD8^+^ T cells is shown in Fig. S3A. Only sGn-H (HSVgB) mRNA-LNP immunized mice showed a statistically significant increase in AIM^+^ CD4^+^ and CD8^+^ T cell populations upon peptide pool stimulation, compared to the unstimulated control (Fig. 4A). In contrast, no significant activation was observed in the sGn-H (human) or PBS groups. These findings indicate that sGn-H (HSVgB) mRNA-LNP vaccination induces more antigen-specific CD4^+^ and CD8^+^ T cell responses.

The activation of CD4^+^ and CD8^+^ T cells and their migration to infection sites are influenced by cytokines and chemokines^55,56^. To assess this, we stimulated splenocytes ex vivo with an sGn-H overlapping peptide pool and analyzed Th1 and Th2 cytokine and chemokine secretion using Olink Proteomics. At week 10, both vaccine groups exhibited significantly higher Th1 cytokines (IL-2, TNF-α, IFN-γ) than the PBS group, with the sGn-H (HSVgB) group showing a stronger and statistically significant increase (Fig. 4B–D). However, no significant difference was observed between the sGn-H (HSVgB) and sGn-H (human) groups. By week 22, the Th1 cytokine levels had declined but remained elevated in the sGn-H (HSVgB) group relative to PBS. In contrast, sGn-H (HSVgB) immunization induced significantly higher levels of Th2 cytokines (IL-4, IL-5, IL-10) at week 10 compared to the PBS and sGn-H (human) groups; like Th1 cytokine levels, Th2 levels declined over time but remained elevated in the sGn-H (HSVgB) group (Fig. 4E–G). These results indicate that sGn-H (HSVgB) immunization elicits a more pronounced Th2-biased response than sGn-H (human).

Additional interleukin (IL) cytokines associated with broader immune activation, including IL-6, IL-22, and IL-3, were also elevated in the sGn-H (HSVgB) group (Fig. S4A). In addition, the sGn-H (HSVgB) group showed increased expression of regulatory cytokines such as CSF2 and PDCD1LG2 (Fig. S4B). Lastly, chemokines CCL2, CCL4, CCL5, CCL22, CXCL2, and CXCL9 were significantly elevated in both vaccine groups compared to PBS, with CCL4 specifically increased in the sGn-H (HSVgB) group (Fig. S4C). Collectively, these data suggest that sGn-H (HSVgB) immunization induces stronger Th2 related cytokine and chemokine responses than sGn-H (human) immunization.

### sGn-H (HSVgB) mRNA-LNPs confer protection in A129 mice

**Fig. 5:**
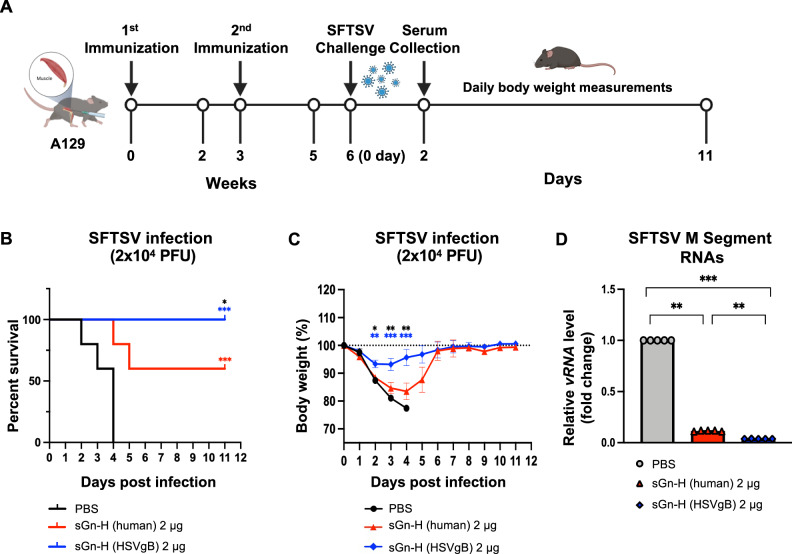
sGn-H (HSVgB) mRNA-LNPs confer protection against SFTSV challenge in A129 mice. **A** Experimental design and timeline of A129 immunization and challenge. Three groups of 6–8-week-old female type I interferon α/β receptor knockout (A129) mice (*n* = 10 per group) were immunized intramuscularly with PBS, 2 μg of sGn-H (human) mRNA-LNP, or 2 μg of sGn-H (HSVgB) mRNA-LNP at weeks 0 and 3. All mice were challenged with a lethal dose of 2 × 10^4^ PFU of SFTSV at week 6. Mouse sera were collected 2 days post-infection (dpi). **B** Mouse survival and **C** body weight changes were monitored daily until 11 dpi. The body weight percentage change was calculated based on the weight at 0 dpi, before the challenge with SFTSV. Statistical analysis was performed between the sGn-H (human) mRNA-LNP and the sGn-H (HSVgB) mRNA-LNP groups. Red asterisks indicate significance between PBS and sGn-H (human) mRNA-LNP; blue asterisks between PBS and sGn-H (HSVgB) mRNA-LNP; black asterisks between sGn-H (human) mRNA-LNP and sGn-H (HSVgB) mRNA-LNP. **D** Viral RNA (vRNA) levels were quantified from sera collected at 2 dpi (*n* = 5 per group) using RT-qPCR. vRNA levels were normalized to *murine gapdh*, and the relative vRNA levels were compared to PBS. Statistical analysis was determined by Mantel-Cox test for survival analysis, by one-way ANOVA with Student’s t-test for weight loss, and by one-way ANOVA with Šídák’s post hoc multiple comparison test for vRNA levels. *p* ≤ 0.05 is denoted by *, *p* ≤ 0.01 by **, *p* ≤ 0.001 by ***, and *ns* indicates no significant difference. **A** image was created with BioRender.com.

To evaluate the protective efficacy of sGn-H vaccines, mRNA-LNP immunization and virus challenge were performed with type I interferon α/β receptor knockout (A129) mice as described in Fig. 5A^57,58^. Due to the impaired innate immune responses in A129 mice, the vaccine dose was increased to 2 µg of mRNA-LNP. Six weeks post-primary immunization, A129 mice were challenged with the SFTSV HB29 strain (2 × 10^4^ PFU), and body weight loss and survival were monitored.

After challenge with a lethal dose of SFTSV, all PBS-immunized mice succumbed to 4 days post-infection (dpi) (Fig. 5B). In contrast, all sGn-H (HSVgB) mRNA-LNP immunized mice survived the duration of the study, while sGn-H (human) mRNA-LNP immunized mice showed a 60% survival rate (Fig. 5B). PBS-immunized mice experienced progressive weight loss exceeding 20% until death, whereas the sGn-H (human) mRNA-LNP group exhibited less than 20% weight loss before recovering to their baseline levels (Fig. 5C). Notably, mice in the sGn-H (HSVgB) mRNA-LNP group lost less than 7% of their baseline body weight by 3 dpi and returned to baseline by 7 dpi. The weight loss pattern closely mirrored survival outcome, further supporting the enhanced protective effect of sGn-H (HSVgB) vaccination. To evaluate virus clearance, serum viral RNA levels were measured at 2 dpi. Both sGn-H (HSVgB) and sGn-H (human) mRNA-LNP immunized groups showed significantly reduced viral RNA levels compared to the PBS control groups, with the sGn-H (HSVgB) group exhibiting lower levels than the sGn-H (human) group (Fig. 5D). Overall, these results indicate that codon optimization of the sGn-H antigen to HSVgB codon usage provides superior protective efficacy compared to human codon usage in mRNA vaccine platform.

## Discussion

In this study, we codon-optimized the SFTSV Gn head (sGn-H) using the codon usage from HSV-1 gB glycoprotein and compared it with a standard human codon-optimized antigen, a commonly used strategy in mRNA vaccine development (Supplementary Table 1). Although the sGn-H (human) and sGn-H (HSVgB) mRNA vaccine constructs encode identical amino acid sequences, they elicited markedly different immunological outcomes. When comparing HSVgB codon usage to human codon usage, notable differences were observed certain amino acids, including serine, proline, and arginine (Supplementary Table 3). For example, HSVgB codon usage favors UCG (serine) and CCG (proline), which are among the least frequently used codon in the human genome. Conversely, AGA, a commonly used codon for arginine in human genome, is rarely used in HSVgB. These differences were incorporated into the codon-optimized sGn-H construct used in this study.

Although the sGn-H vaccine constructs were optimized for human or HSVgB codon usage, their immunogenicity and protective efficacy were evaluated in mice. To assess the relevance of this approach, we compared codon usage frequencies between humans and mice and found that overall patterns are similar (Supplementary Table 3^59^). Based on this similarity, we hypothesized that HSVgB codon optimization could also enhance protein expression in mouse models. To test this, we measured sGn-H accumulation and secretion in human (HEK293T) and mouse (RAW264.7) cells and observed higher levels from sGn-H (HSVgB) in both cell types, supporting the functional relevance of HSVgB codon usage even in non-human hosts. While codon usage frequency provides useful information for comparing translational efficiency, other factors, such as tRNA abundance and mRNA properties, may also contribute to observed differences in protein expression.

BALB/c mice immunized with sGn-H (HSVgB) mRNA-LNP exhibited higher total IgG and neutralizing antibody titers at all time points compared to those immunized with sGn-H (human) mRNA-LNP. Notably, unlike SARS-CoV-2 mRNA vaccines, where antibody titers decline over time, the antibody responses induced by our sGn-H mRNA vaccine remained sustained through week 20^60^. At week 10, we also observed a greater number of sGn-H-specific ASCs in the bone marrow of sGn-H (HSVgB) mRNA-LNP mice. Given that the presence of long-lived plasma cells (LLPCs) in bone marrow has been linked to durable antibody production, this suggests that HSVgB codon optimization may promote sustained and long-lasting humoral immunity^61^. Furthermore, previous studies have shown that SFTSV primarily resides in plasma cells within peripheral blood mononuclear cells (PBMCs) of infected patients, and fatal cases often fail to elicit Gn-specific IgG responses^62,63^. Thus, the ability of sGn-H (HSVgB) mRNA vaccines to elicit sGn-H-specific ASCs in the bone marrow may indicate a protective immune response capable of preventing severe disease. However, additional experiments are needed to determine whether these ASCs are indeed LLPCs. In addition, while overall B cell subset populations were similar between the two vaccine groups at week 10, it remains unclear whether differences exist in sGn-H-specific memory and effector B cell responses. To address this, we plan to perform sGn-H-specific B cell immunophenotyping to further characterize the vaccine-induced B cell compartments.

We also assessed T cell-mediated immune responses by stimulating splenocytes with sGn-H overlapping peptides. In mice immunized with sGn-H (HSVgB) vaccine, we observed increased populations of sGn-H-specific CD4^+^ and CD8^+^ T cells, along with prominently elevated levels of Th2-associated cytokines. As Th2 cytokines promote B cell proliferation, differentiation, and isotype class switching, this likely contributed to the enhanced total IgG and neutralizing antibody responses observed in the sGn-H (HSVgB)-immunized mice^64,65^. Although BALB/c mice are inherently Th2-skewed, the greater Th2 cytokine induction and higher total IgG and neutralizing antibody levels seen with sGn-H (HSVgB) suggest sGn-H (HSVgB) further amplified Th2-biased immune responses.

Due to the impaired innate immune responses in A129 mice, we initially increased the mRNA vaccine dose to 3 μg in our challenge studies. In a previous study with sGn-H (human)-ferritin fusion mRNA-LNP vaccine, this dose resulted in 100% survival following challenge of A129 mice with a lethal dose of 2 × 10^4^ PFU^21^. In the present study, we reduced the mRNA vaccine dose to 2 μg to assess whether effective protection could still be achieved at a lower dose. At this lower dose, mice immunized with sGn-H (human) mRNA-LNP showed substantial weight loss and only a 60% survival rate, whereas all mice immunized with sGn-H (HSVgB) mRNA-LNP survived with reduced serum viral RNA levels. These results indicate that mRNA dosage critically influences vaccine efficacy and that HSVgB codon optimization can enhance protection, even at reduced dosing.

While HSVgB codon optimization led to higher protein expression, enhanced immune responses, and improved protection, the underlying mechanism remains unclear. One possibility is that codon optimization influences mRNA-level properties. For example, increased GC content has been associated with greater mRNA stability and protein expression^66^. The GC content of the sGn-H (human) mRNA was 61.3%, compared to 66.8% for sGn-H (HSVgB), closely matching the native HSVgB gene (66.6%) (Supplementary Table 1). To assess mRNA stability, we performed an RT-qPCR time course analysis following mRNA transfection. Although sGn-H (HSVgB) mRNA levels were slightly higher over time compared to sGn-H (human) mRNAs, the differences were not statistically significant (Fig. S5). We also analyzed dinucleotide frequencies, focusing on CpG and UpA motifs, which are known to affect mRNA stability and trigger innate immune pathways, such as ZAP-mediated degradation and RNase L activation (Supplementary Table 2^67^). Both CpG and UpA motifs were under-represented: CpG observed/expected (O/E) ratios were 0.625 for sGn-H (human) and 0.795 for sGn-H (HSVgB) and UpA ratios were 0.398 and 0.379, respectively, indicating no substantial differences that would suggest altered immune activation between human and HSVgB constructs.

Given that these analyses revealed no major differences in mRNA stability or dinucleotide composition, the enhanced performance of sGn-H (HSVgB) may instead arise from effects on translation efficiency or co-translational folding. Ribosome profiling, as previously suggested^46^, could help determine whether codon usage influences ribosome density, pausing, or elongation kinetics. We also considered the potential role of RNA modifications, such as m⁶A, which can impact mRNA fate and translation; however, preliminary analyses did not reveal significant differences in m⁶A patterns between the two constructs (data not shown). Collectively, these results suggest that HSVgB codon optimization offers benefits that cannot be fully attributed to mRNA stability or innate immune sensing, instead implicating differences in translational dynamics or post-translational mechanisms as likely contributors.

Because sGn-H (human) and sGn-H (HSVgB) mRNAs encode identical amino acid sequences and differ only in codon usage, we hypothesize that the observed differences also arise at the translation level. Codon usage can affect translation elongation speed and co-translational folding, which in turn affect protein expression and antigen quality^34^. To investigate this, we plan to employ ribosome profiling, a technique that maps ribosome positions across mRNAs and provides insights into translation rates^68^. Previous studies have demonstrated that preferred codons can accelerate translation, while rare codons may cause ribosome stalling and slow translation^69,70^. Notably, the sGn-H (HSVgB) contains a higher proportion of rare codons, such as UCG (Ser) and CCG (Pro), than sGn-H (human), which may impact co-translational folding and enhance immunogenicity. These insights will help elucidate the translational mechanisms by which codon usage influences vaccine efficacy.

In conclusion, our results demonstrate that HSVgB codon optimization of the sGn-H antigen enhances the immunogenicity and protective efficacy of SFTSV mRNA vaccines, even at reduced doses. These findings highlight the potential of viral codon optimization strategies to improve the design and effectiveness of mRNA vaccines.

## Methods

### Cells and viruses

HEK293T, RAW264.7, and Vero E6 cells were purchased from ATCC and cultured in Dulbecco’s Modified Eagle Medium (DMEM) containing 10% fetal bovine serum (FBS) and 1% penicillin/streptomycin in a 5% CO_2_ incubator at 37 °C. The SFTSV Hubei 29 (HB29) strain (GenBank accessions KP202163 to KP202165) was propagated by infecting confluent Vero E6 cells, and the supernatant was harvested at 7 dpi. The viral titer was determined using a plaque assay, in which the serially diluted virus infected confluent Vero E6 cells, and the cells were incubated for 7 days in overlay media consisting of DMEM with 1% UltraPure™ Low Melting Point Agarose (Invitrogen). The monolayers were fixed with 4% formaldehyde and stained with 0.2% crystal violet in 20% ethanol to count plaque numbers in PFU/mL.

### SFTSV Gn-H vaccine construct design

The SFTSV Gn-H domain (residues 21–340 of the Gn glycoprotein; GenBank: MG737175.1) was selected based on previously published papers^21,27^. The gene was codon-optimized for human codon usage or HSV-1 glycoprotein B (HSVgB) (Gene ID: 24271469) codon usage. These genes were optimized and synthesized by GenScript. The viral signal peptide was replaced with the Lucia^®^ luciferase signal peptide. The sequences were then cloned into an in-house-generated pZMV vector, which contains a T7 promoter and 5′ and 3′ untranslated regions (UTRs)^21^.

### SFTSV Gn-H mRNA generation

mRNA production by in vitro transcription (IVT) was performed through co-capping IVT using the HiScribe^®^ T7 High Yield RNA Synthesis Kit (New England Biolabs) and CleanCap® Reagent AG (3’ OMe) (TriLink). During this process, uridine triphosphate (UTP) was 100% replaced with N^1^-methyl-pseudoUTP (TriLink). Poly(A) tails were enzymatically added to the 3′ end of IVT RNA products using *E. coli* poly(A) polymerase (NEB). sGn-H mRNAs were purified using a spin column (Zymo Research), and the poly(A) tail addition was verified using a 1.5% agarose gel in 3-(N-morpholino)propanesulfonic acid (MOPS) buffer.

### DNA and mRNA transfection

For DNA transfection, 1 μg of sGn-H-encoding plasmids was transfected into HEK293T cells using PolyJet transfection reagent (SignaGen). Cell lysates were collected 48 h post-transfection. For mRNA transfection, 2.5 μg of sGn-H mRNA was transfected into HEK293T and RAW264.7 cells using TransIT-mRNA transfection reagents (Mirus). After transfection, the culture media was replaced with FreeStyle™ 293 Expression Medium for HEK293T cells and serum-free DMEM for RAW264.7 cells. At 24 h post-transfection, the supernatant was collected and proteins were precipitated by adding 100% trichloroacetic acid (TCA) at a 1:10 (v/v) ratio. Samples were incubated on ice for 30 min and centrifuged at 10,000 × *g* for 10 min at 4 °C. The protein pellets were washed twice with 500 μL of ice-cold acetone at the same centrifugation conditions. Precipitated proteins were resuspended in 6x Laemmli SDS-sample buffer (bioWORLD). Cell lysates were also collected 24 h post-transfection. Total protein concentrations from the cell lysates were quantified using the Bradford assay.

### Lipid nanoparticle (LNP) generation and transfection

sGn-H mRNAs and GenVoy-ILM™ lipid (Cytiva) were mixed in a 3:1 ratio (mRNA:lipid, v/v) using a NanoAssemblr Ignite+ microfluidic instrument (Precision Nanosystems). mRNA-encapsulated LNPs (mRNA-LNPs) were diluted in phosphate-buffered saline (PBS) and concentrated using Amicon^®^ Ultra centrifugal filters (Millipore). The mRNA concentration and encapsulation efficiency were measured using the Quant-iT™ RiboGreen RNA assay (ThermoFisher). 1 μg of mRNA-LNPs, along with human apolipoprotein E (Millipore Sigma), were transfected into HEK293T and RAW264.7 cells. Subsequent steps were identical to those described for mRNA transfection.

### Western blot

Cell lysate and supernatant samples were mixed with 6x Laemmli SDS-sample buffer (bioWORLD), boiled at 95 °C for 5 min, and loaded onto SDS-PAGE gels for protein separation prior to Western blotting. Western blotting was performed using an in-house-generated anti-SFTSV Gn antibody, followed by an HRP-conjugated anti-mouse IgG secondary antibody (Cell Signaling). Anti-GAPDH antibody (0411) (Santa Cruz Biotechnology) and anti-β-actin antibody (C4) (Santa Cruz Biotechnology) were used as internal loading controls, followed by the same HRP-conjugated anti-mouse IgG antibody.

### Mice

Six- to eight-week-old female BALB/c mice and 6–8-week-old type I interferon α/β receptor knockout (A129) mice were purchased from The Jackson Laboratory and the Mutant Mouse Resource and Research Center (MMRRC) at The Jackson Laboratory, respectively. All mouse studies were conducted in accordance with protocols approved by the Cleveland Clinic Institutional Animal Care and Use Committee (IACUC). mRNA-LNP immunizations were performed in the standard ABSL-2 animal facility, while SFTSV challenges were conducted in the ABSL-3 facility at the Cleveland Clinic.

### Mouse immunization

Female BALB/c mice were immunized with PBS or 1 μg of sGn-H (human) or sGn-H (HSVgB) mRNA-LNPs via intramuscular injection at weeks 0 and 3. Mouse blood was collected at various time points via the retro-orbital route, and sera were separated and heat-inactivated at 55 °C for 30 min. Prior to each immunization and blood collection, mice were anesthetized with 3–4% isoflurane delivered via inhalation using a SomnoFlo^®^ Low-Flow Electronic Vaporizer (Kent Scientific)^71^. For organ harvest, mice were euthanized by CO_2_ asphyxiation followed by cervical dislocation. Spleens were harvested at weeks 10 and 22, and bone marrow cells were collected at week 10 for subsequent analyses.

### Mouse challenge with SFTSV HB29

Female A129 mice were immunized with PBS or 2 μg of sGn-H (human) or sGn-H (HSVgB) mRNA-LNPs via intramuscular injection at weeks 0 and 3. At week 6 post-primary immunization, mice were challenged with a lethal dose (2 × 10^4^ PFU) of live SFTSV HB29 via intramuscular injection. Prior to immunization and viral challenge, mice were anesthetized with 3–4% isoflurane delivered via inhalation using a SomnoFlo^®^ Low-Flow Electronic Vaporizer (Kent Scientific)^71^. Mice were monitored daily for changes in body weight and clinical signs. Sera were collected at 2 dpi for viral load analysis. Mice were euthanized by CO_2_ asphyxiation followed by cervical dislocation, either upon reaching humane endpoint criteria based on clinical scoring (e.g., >20% weight loss or reduced physical activity), or at the end of the study.

### Enzyme-linked immunosorbent assay (ELISA)

The SFTSV Gn-H protein, purified as described in our published paper, was coated onto Immulon 4 HBX 96-well plates (ThermoFisher) at a concentration of 1 µg/mL^27^. The plates were blocked with 3% bovine serum albumin (BSA) in PBS at 4 °C. Following washing with 0.05% Tween-20 in PBS (PBS-T), serially diluted, heat-inactivated mouse sera were added, and the plates were incubated overnight at 4 °C. After washing the plates with PBS-T, an HRP-conjugated anti-mouse IgG antibody (Cell Signaling) was used as the secondary antibody. After the final wash, 3,3′,5,5′-Tetramethylbenzidine dihydrochloride (TMB) 2-Component Microwell Peroxidase Substrate (SeraCare) was added, and the reaction was terminated by adding 2 N sulfuric acid. The endpoint titer was calculated as the dilution at which the absorbance exceeded three times background absorbance.

### Pseudovirus neutralization assay

A recombinant vesicular stomatitis virus (rVSV) pseudovirus expressing SFTSV Gn/Gc from the HB29 strain and a luciferase reporter (rVSV-SFTSV G-Luc) was generated using previously described protocols^21,27^. The rVSV-SFTSV G-Luc pseudovirus was harvested 24 h post-infection, filtered through a 0.45 μm filter, and stored as aliquots at −80 °C for subsequent neutralization assays. For the neutralization assay, heat-inactivated sera were serially diluted in DMEM and incubated with rVSV-SFTSV G-Luc for 1 h at 37 °C. The virus-serum mixtures were then added to HEK293T cells and incubated for 24 h at 37 °C to allow for infection. Infected cells were lysed, and the luciferase activity was measured in relative light units (RLU) using the Luciferase Assay System (Promega). Neutralization activity was calculated by normalizing the RLU values: wells with no serum (infected with rVSV-SFTSV G-Luc without serum) were set as 0% neutralization, and wells with no pseudovirus (media only) were set as 100% neutralization. IC_50_ values were determined by fitting the neutralization curves using a log (agonist) vs. normalized response (variable slope) nonlinear regression model in GraphPad Prism v10.

### B-cell immunophenotyping assay

The B-cell immunophenotyping assay was adapted to detect surface markers on B cells from the spleen, as adopted from published papers^72^. Splenocytes from BALB/c mice immunized with PBS, sGn-H (human) mRNA-LNP, or sGn-H (HSVgB) mRNA-LNP were harvested at week 10 post-primary immunization. 2 × 10^6^ splenocytes per well were seeded into a 96-well V-bottom plate (Corning). The cells were blocked with anti-CD16/CD32, TruStain FcX™ PLUS (BioLegend) in FACS buffer (PBS with 2% heat-inactivated FBS) and incubated at 4 °C for 15 min. B-cell immunophenotyping antibody cocktails, in the presence of LIVE/DEAD™ Fixable Blue Dead Cell Stain (Invitrogen), True-Stain Monocyte Blocker (BioLegend), and Brilliant Stain Buffer Plus (BD Biosciences) were used to stain the cells for an additional 30 min at 4 °C. Unstained cells were included as a negative control. After washing with FACS buffer, the stained cells were fixed with 4% paraformaldehyde for 15 min at room temperature in the dark. The cells were washed and resuspended in 100 µL of FACS buffer containing a Tandem Stabilizer (BioLegend). Cells were stored at 4 °C in the dark prior to acquisition on a SONY ID7000 Spectral Cell Analyzer (Sony Biotechnology). Flow cytometric data were analyzed using FlowJo (v10.9.0, Treestar Inc.). The upstream gating strategy is shown in Fig. S1A. All antibodies and reagents used in this assay are listed in Table 1.

**Table 1:** Staining cocktail and dilutions for B cell Immunophenotyping

Marker	Fluorochrome	Company	Clone	Dilution
CD19	BV605	BioLegend	6D5	1:100
CD23	PE	BioLegend	B3B4	1:200
CD138	PE/Fire810	BioLegend	281-2	1:100
CD93	APC	BioLegend	AA4.1	1:100
CD274	BV650	BioLegend	10F.9G2	1:100
CD205	APC/Fire750	BioLegend	NLDC-145	1:800
B220	BV750	BioLegend	RA3-6B2	1:100
GL7	BV421	BD Biosciences	GL7	1:200
CD69	Alexa 488	BioLegend	H1.2F3	1:100
Fixable Live/Dead	Live/Dead Fix Blue	Thermo Fisher Scientific		0.2 µl/sample
True-Stain Monocyte Blocker™		BioLegend		5 µl/sample
BD Horizon™ Brilliant Stain Buffer Plus		BD Biosciences		10 µl/sample

### Enzyme-linked immunospot (ELISpot) assay

The ELISpot assay was adapted to detect sGn-H-specific antibody-secreting cells (ASCs) from mouse bone marrow, based on methods described in previous publications^73,74^. Multiscreen^®^ 96-well plates, hydrophobic PVDF membranes (Millipore) were pre-wetted with 15 µL of 35% ethanol for less than 1 min and immediately washed with PBS. Plates were then coated with 100 µL of 5 µg/mL of purified sGn-H protein and incubated overnight at 4 °C. Following another PBS wash, plates were blocked for 2 h at room temperature with RPMI 1640 + 10% FBS. 2.1 × 10^6^ bone marrow cells were serially diluted twofold and then seeded into the plates. Plates were incubated at 37 °C for 6 h, then transferred to 4 °C for overnight incubation. After washing with PBS-T, 0.5 µg/mL of biotinylated Jackson Immuno Rabbit Anti-Mouse IgG detection antibody (Jackson ImmunoResearch) in PBS + 5% FBS was added to each well and incubated for 2 h at room temperature. Detection of sGn-H-specific ASCs was performed using a streptavidin-alkaline phosphatase conjugate (Invitrogen) and the BCIP (5-bromo-4-chloro-3-indolylphosphate)/NBT substrate (Roche). The images of wells were taken from CTL ImmunoSpot S6 Universal-V Analyzer (Cellular Technology Ltd.).

### Multiplexed activation-induced marker (AIM) assay

The multiplexed AIM assay was adapted to detect sGn-H-specific CD4^+^ and CD8^+^ T cells, as described in previous publications^75–81^. Splenocytes from BALB/c mice immunized with PBS, sGn-H (human) mRNA-LNP, or sGn-H (HSVgB) mRNA-LNP were harvested at week 10 post-primary immunization. 2 × 10^6^ splenocytes per well were seeded into a 96-well V-bottom plate (Corning), and 0.5 µg/mL of CD40-blocking antibody was added, followed by a 15-min incubation. The cells were then stimulated with 1 µg/mL of the sGn-H overlapping peptide pool (15-mer with 11 amino acid overlap, GenScript) for 20 h at 37 °C. Unstimulated wells served as negative controls for each sample. After overnight stimulation, cells were washed twice with FACS buffer (PBS with 2% heat-inactivated FBS and 0.5 mM EDTA), incubated with anti-CD16/CD32, TruStain FcX™ PLUS (BioLegend) for 15 min, and then stained for 30 min with AIM assay antibody cocktail in the presence of True-Stain Monocyte Blocker (BioLegend) and Brilliant Stain Buffer Plus (BD Biosciences). The stained cells were fixed with 4% paraformaldehyde for 10 min at room temperature. The cells were washed and resuspended in 100 µL of FACS buffer containing a Tandem Stabilizer (BioLegend). Cells were stored at 4 °C in the dark prior to acquisition on a SONY ID7000 Spectral Cell Analyzer (Sony Biotechnology). Flow cytometric data were analyzed using FlowJo (v10.9.0, Treestar Inc.). The upstream gating strategy is shown in Fig. S3A. All antibodies and reagents used in this assay are listed in Table 2.

**Table 2:** Staining cocktail and dilutions for AIM assay

Marker	Fluorochrome	Company	Clone	Dilution	Note
CD11b	BV711	BioLegend	M1/70	1:200	Dump
CD19	BV711	BioLegend	6D5	1:200	Dump
CD3	BV785	BioLegend	17A2	1:100	
CD4	APC-eF780	Thermo Fisher Scientific	RM4-5	1:400	
CD8a	eF450	Thermo Fisher Scientific	53–6.7	1:200	
CD69	BV510	BD Biosciences	H1.2F3	1:100	
CD137	PerCP-eFluor710	Thermo Fisher Scientific	17B5	1:200	
CD134	APC	BioLegend	OX-86	1:100	
CD40L	FITC	BioLegend	SA047C3	1:100	
Fixable Live/Dead	Live/Dead Fix Blue	Thermo Fisher Scientific		0.2 µl/sample	
True-Stain Monocyte Blocker™		BioLegend		5 µl/sample	
BD Horizon™ Brilliant Stain Buffer Plus		BD Biosciences		10 µl/sample	

### Olink proteomics

Splenocytes from BALB/c mice immunized with PBS, sGn-H (human) mRNA-LNP, or sGn-H (HSVgB) mRNA-LNP were harvested at weeks 10 and 22 post-primary immunization. 2 × 10^5^ mouse splenocytes per well were seeded into a 96-well clear V-bottom plate (Corning). The cells were ex vivo stimulated with 1 μg/mL of the same sGn-H overlapping peptide pool used in the AIM assay for 24 h at 37 °C. After centrifuging the plates at 400 × *g* for 7 min at 4 °C, the supernatant was collected and transferred to a 96-well clear flat-bottom Ultra-Low Attachment plate (Corning). The samples were run on Olink^®^ Signature Q100 using the Olink^®^ Target 48 Mouse Cytokine panel to detect cytokine and chemokine concentrations.

### Viral RNA extraction and quantitative PCR (qPCR) for viral RNA detection

Viral RNAs were extracted from the sera of A129 mice at 2 dpi using TRIzol reagent (ThermoFisher Scientific) according to the manufacturer’s protocol. RNA concentration and purity were assessed using a Nanodrop spectrophotometer (ThermoFisher Scientific), and samples with a 260/280 ratio between 1.8 and 2.0 were used for qPCR analysis. For qPCR, complementary DNA (cDNA) was synthesized from 1 µg of extracted RNA using an iScript cDNA Synthesis Kit (Bio-Rad), following the manufacturer’s protocol. The qPCR assay was conducted using the SsoAdvanced Universal SYBR Green Master Mix (Bio-Rad) on a CFX96 Real-Time System (Bio-Rad). Primers targeting the SFTSV M segment were obtained from published literature and are listed in Table 3^27^. The thermocycling conditions were as follows: denaturation at 95 °C for 3 min, followed by 40 cycles of denaturation at 95 °C for 10 s, annealing at 60 °C for 10 s, and extension at 72 °C for 30 s. Relative quantification of viral RNA was performed using the comparative Ct (ΔΔCt) method. Ct values were normalized to the expression of a reference gene (murine *Gapdh*), and fold changes in viral RNA levels were calculated relative to the control group (PBS-immunized mice).

**Table 3:** Primers for RT-qPCR

SFTSV M Segment	
Primer Names	Sequences
SFTSV M segment RT-qPCR FWD	5′-AATTCACATTTGAGGGTAGTT-3′
SFTSV M segment RT-qPCR REV	5′-TATCCAAGGAGGATGACAATAAT-3′
Murine *Gapdh* RT-qPCR FWD	5′-GCCTCTCTTGCTCAGTGTCC-3′
Murine *Gapdh* RT-qPCR REV	5′-CTCCCACTCTTCCACCTTCG-3′
**sGn-H mRNA Stability**	
Primer Names	Sequences
sGn-H RT-qPCR FWD	5′-TGTTCAACCAGTGCGAGGGC-3′
sGn-H RT-qPCR REV	5′-TGGTCTTGTGCTCGCGCATG-3′
18 s rRNA (human) RT-qPCR FWD	5′-TTCGAACGTCTGCCCTATCAA-3′
18 s rRNA (human) RT-qPCR REV	5′-GATGTGGTAGCCGTTTCTCAGG-3′

### Reverse transcription (RT)-qPCR for sGn-H mRNA stability

2.5 μg of sGn-H mRNA was transfected into HEK293T cells using TransIT-mRNA transfection reagent (Mirus). At 6 h post-transfection (hpt), the transfected cells were detached and reseeded for each subsequent time point (12, 24, 36, and 48 hpt). At the indicated time points, cells were harvested. Total cellular RNA was extracted using the PureLink^TM^ RNA Mini Kit (Invitrogen) according to the manufacturer’s protocol. 1 µg of total RNA was treated with DNase I (Sigma-Aldrich), and then reverse transcribed into cDNA using an iScript cDNA Synthesis Kit (Bio-Rad). The qPCR was conducted using the SsoAdvanced Universal SYBR Green Master Mix (Bio-Rad) on a CFX96 Real-Time System (Bio-Rad). Primers targeting sGn-H are listed in Table 3. The thermocycling conditions were as follows: initial denaturation at 95 °C for 30 s, followed by 40 cycles of 95 °C for 10 s and 60 °C for 30 s. Relative quantification of sGn-H mRNA levels was performed using the comparative Ct (ΔΔCt) method. Ct values were normalized to human 18 s rRNA, and fold changes were calculated relative to the 6-h time point.

### Statistical analyses

Statistical analyses were conducted using Microsoft Excel or GraphPad Prism v10. One-way analysis of variance (ANOVA) with Šídák’s post hoc multiple comparison test was used for DNA transfection experiments. For mRNA and LNP transfection experiments, Student’s t-test was performed. For sGn-H-specific IgG antibody titers and neutralization assays against SFTSV, Student’s t-test was applied. Data are expressed as geometric means with 95% confidence intervals (CI). One-way ANOVA with Šídák’s post hoc multiple comparison test was used for B cell immunophenotyping, ELISpot assay, Olink proteomics, and qPCR analyses. Two-way ANOVA with Šídák’s post hoc multiple comparison test was performed for T cell AIM assay. These data are presented as mean values with error bars indicating standard error of mean (SEM). Differences in survival curves were analyzed using the Mantel-Cox test. Student’s t-test was applied for mRNA stability test. Statistical significance thresholds were set as follows: *p ≤ 0.05, **p ≤ 0.01, ***p ≤ 0.001, ****p ≤ 0.0001, ns, not significance.

## Supplementary information

Supplementary Information

## Data Availability

Data supporting this study are available from the corresponding author upon reasonable request.
