# Supplementary material for: A viral codon usage strategy enhances antigen production and protection in SFTSV mRNA vaccination
Source: NPJ Vaccines. 2025 Nov 24;10:248. doi: 10.1038/s41541-025-01298-4 (PMC12644672; doi:10.1038/s41541-025-01298-4)
Supplement: Supplementary file 1 — Supplementary Information [file 41541_2025_1298_MOESM1_ESM.pdf]

Supplementary Figure 1

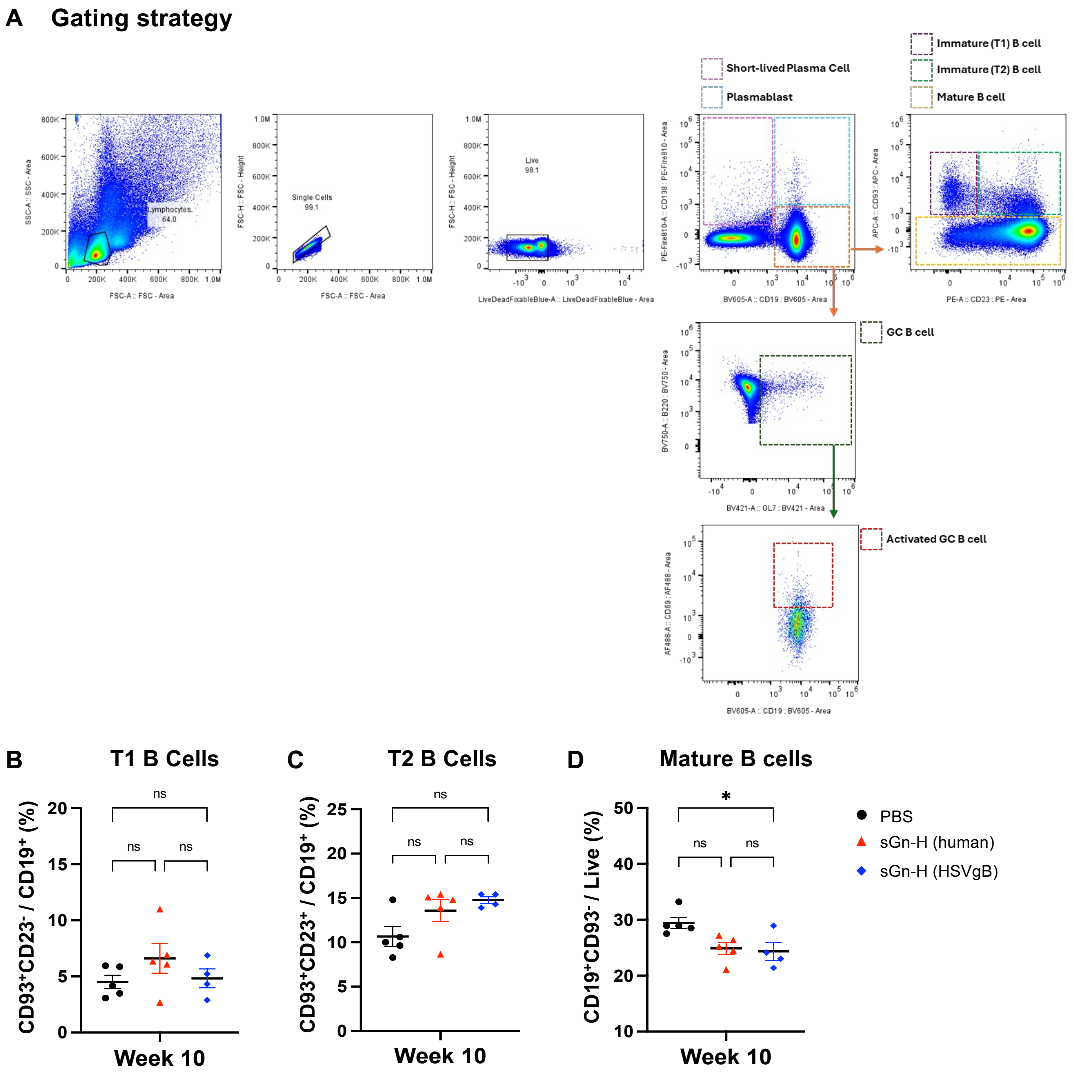

Supplementary Figure 2

A

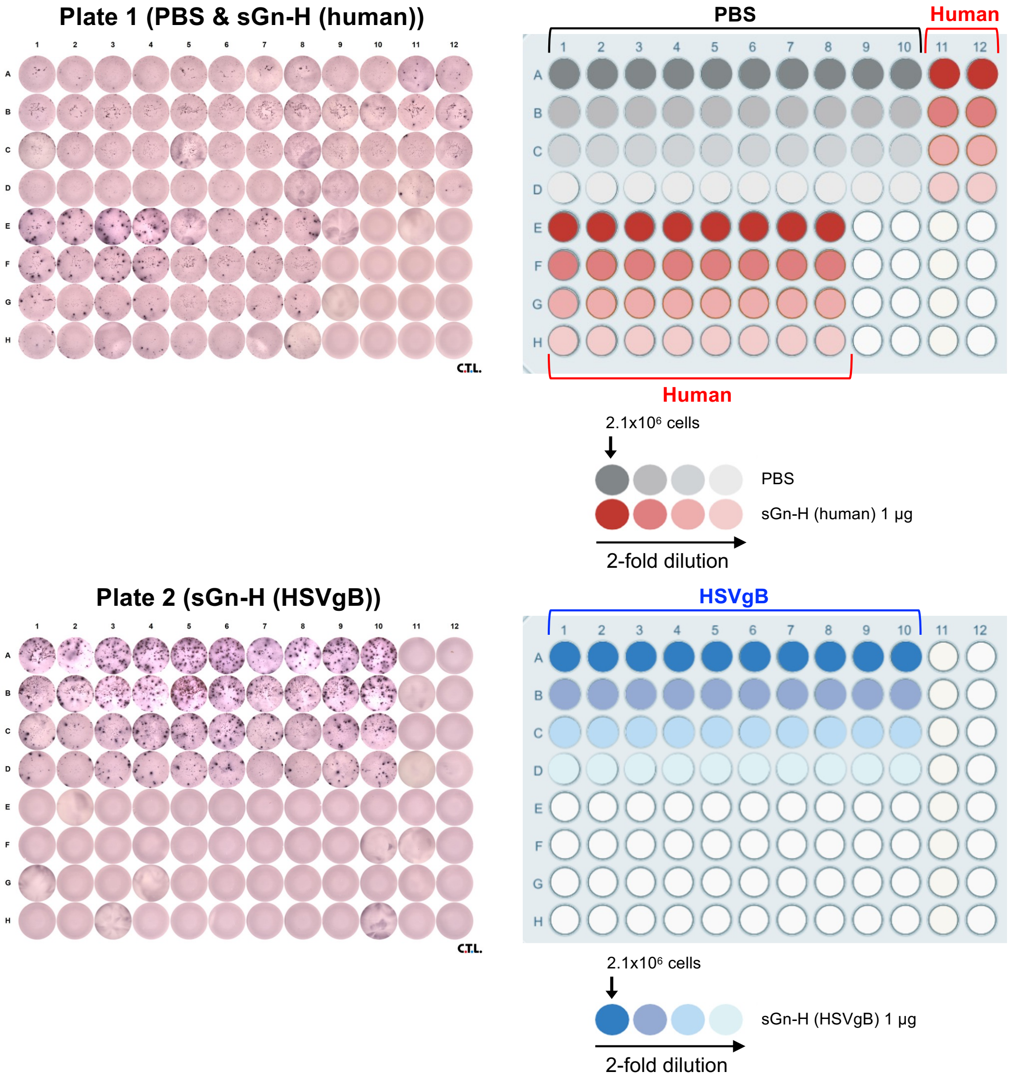

Supplementary Figure 3

A Gating strategy

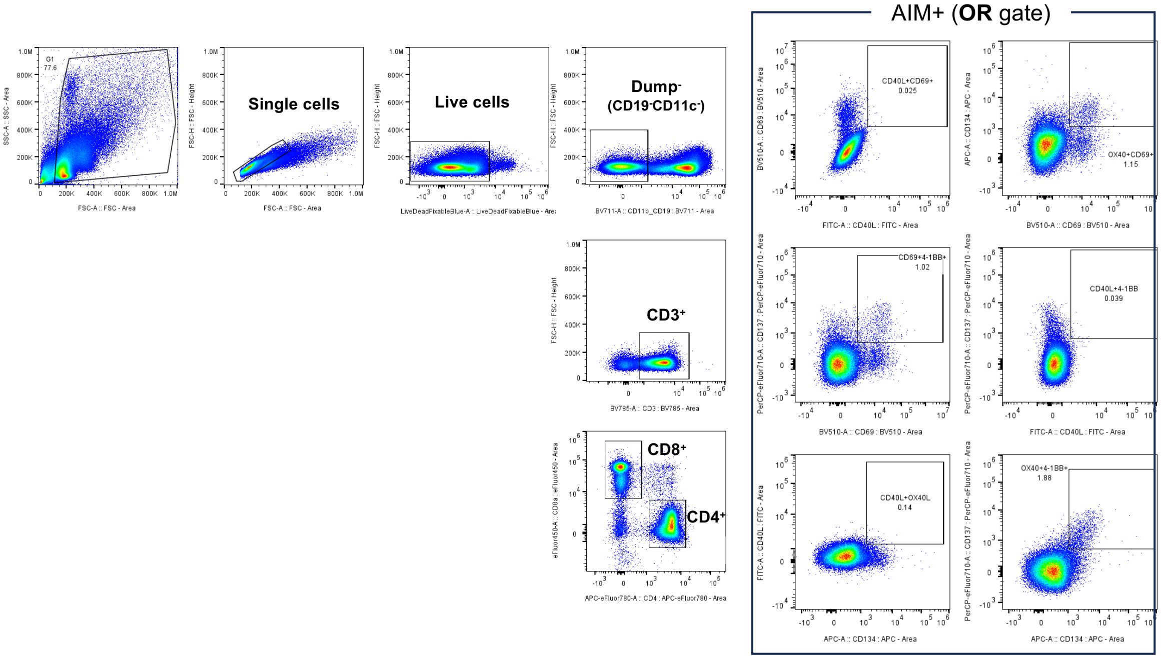

Supplementary Figure 4

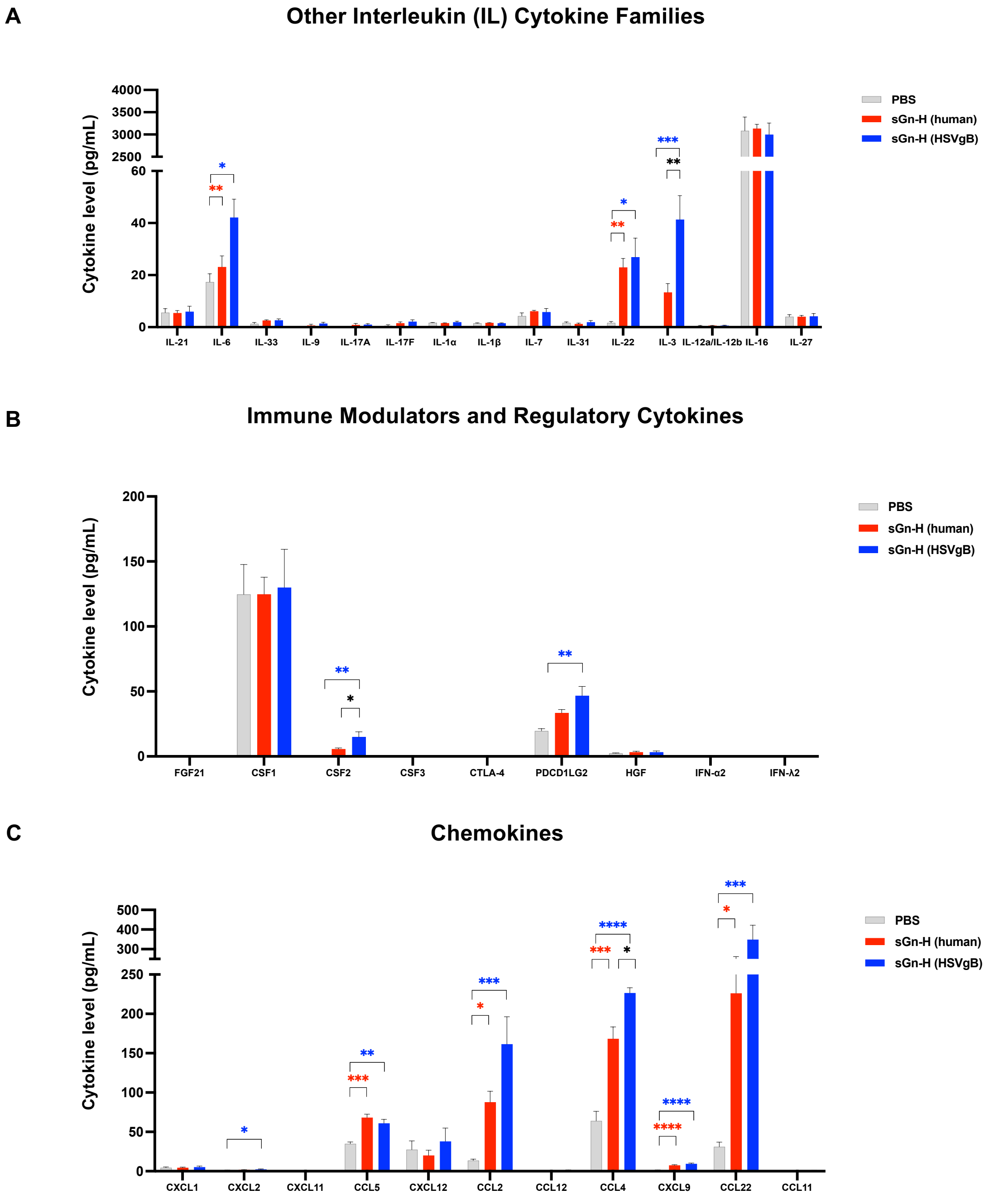

Supplementary Figure 5

A

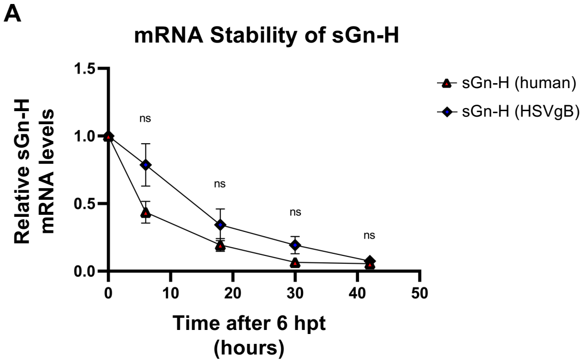

Supplementary Table 1. Full sGn-H mRNA Sequences

sGn-H (human) sequence (960 bps, 320 AA, GC content: 61.3%)

ACCGGTCCTATCATCTGTGCCGGACCCATCCACAGCAACAAGAGCGCCGACATCCCCCATC
TGCTGGGCTACAGCGAGAAGATTTGCCAGATCGACCGGCTGATCCACGTGTCCTCTTGGCT
GCGGAACCACAGCCAGTTCCAGGGCTACGTGGGACAGAGAGGCGGCAGATCCCAGGTGT
CCTACTACCCAGCTGAAAACAGCTACAGCCGGTGGAGCGGCCTGCTGAGCCCTTGTGATG
CTGACTGGCTGGGCATGCTGGTCGTGAAGAAGGCCAAGGGCAGCGACATGATCGTGCCTG
GCCCTAGCTACAAGGGCAAGGTGTTCTTCGAGCGGCCCACCTTCGACGGCTATGTGGGCT
GGGGATGTGGCAGCGGCAAGAGCAGAACAGAGTCCGGCGAGCTGTGCAGCAGCGATTCT
GGCACAAGCTCTGGCCTGCTGCCCAGCGACAGAGTGCTGTGGATTGGCGACGTGGCCTG
CCAGCCCATGACCCCTATCCCCGAGGAAACCTTTCTGGAACTGAAGTCCTTCAGCCAGAGC
GAGTTCCCCGACATCTGCAAGATCGACGGCATCGTGTTCAACCAGTGCGAGGGCGAGAGC
CTGCCCCAGCCTTTTGATGTGGCCTGGATGGACGTGGGCCACTCCCACAAGATCATCATGC
GCGAGCACAAGACCAAATGGGTGCAGGAAAGCAGCAGCAAGGACTTCGTGTGCTACAAAG
AGGGCACCGGCCCCTGCAGCGAGAGCGAGGAAAAGACCTGCAAGACCAGCGGCTCCTGC
AGAGGCGACATGCAGTTCTGCAAGGTGGCCGGATGCGAGCACGGCGAAGAGGCCAGCGA
GGCCAAGTGCAGATGTAGCCTGGTGCACAAGCCCGGCGAGGTGGTGGTGTCTTACGGCG
GAATGAGAGTGCGGCCCAAGTGCTACGGCTTCAGCCGGATGATGGCCACCCTGGAAGTGA
AC

sGn-H (HSVgB) sequence (960 bps, 320 AA, GC content: 66.8%)

ACCGGGCCGATCATCTGCGCCGGGCCGATCCACTCCAACAAGTCCGCCGACATCCCCCAC
CTGCTGGGCTACTCCGAGAAGATCTGCCAGATCGACCGCCTGATCCACGTGTCCAGCTGG
CTGCGCAACCACTCGCAGTTCCAGGGCTACGTGGGCCAGCGCGGTGGCCGCTCCCAGGT
GAGCTACTACCCGGCCGAGAACAGCTACTCGCGCTGGTCGGGCCTGCTGAGCCCCTGCG
ACGCCGACTGGCTGGGCATGCTGGTGGTGAAGAAGGCCAAGGGCTCGGACATGATCGTG
CCGGGCCCCAGCTACAAGGGCAAGGTGTTCTTCGAGCGCCCCACCTTCGACGGCTACGTG
GGCTGGGGCTGCGGCTCCGGCAAGTCCCGCACCGAGTCGGGCGAGCTGTGCTCCTCGG
ACTCGGGCACCTCCTCGGGCCTGCTGCCCTCGGACCGCGTGCTGTGGATCGGCGACGTG
GCCTGCCAGCCCATGACCCCCATCCCGGAGGAGACGTTCCTGGAGCTGAAGAGCTTCTCC
CAGAGCGAGTTCCCCGACATCTGCAAGATCGACGGCATCGTGTTCAACCAGTGCGAGGGC
GAGAGCCTGCCCCAGCCCTTCGACGTGGCCTGGATGGACGTGGGCCACAGCCACAAGAT
CATCATGCGCGAGCACAAGACCAAGTGGGTGCAGGAGTCCAGCTCCAAGGACTTCGTGTG
CTACAAAGAGGGCACCGGCCCCTGCTCGGAGTCGGAGGAGAAGACCTGCAAGACCTCGG
GCAGCTGCCGCGGCGACATGCAGTTCTGCAAGGTGGCCGGCTGCGAGCACGGCGAGGAA
GCCTCGGAGGCCAAGTGCCGCTGCTCCCTGGTGCACAAGCCGGGCGAGGTGGTGGTGTC
CTACGGTGGCATGCGCGTGCGCCCGAAGTGCTACGGCTTCAGCCGCATGATGGCCACCCT
GGAGGTGAAC

Supplementary Table 2. Dinucleotide Counts and Observed/Expected Frequency Ratios in sGn-H Sequences

	sGn-H (human)		sGn-H (HSVgB)	
**Dinucleotide**	**Observed Count**	**Observed / Expected (O/E) Frequency**	**Observed Count**	**Observed / Expected (O/E) Frequency**
**AA**	42	0.881	28	0.942
**AC**	48	0.778	50	0.886
**AG**	90	1.300	67	1.191
**AU**	34	0.966	24	0.910
**CA**	78	1.265	63	1.116
**CC**	84	1.052	103	0.961
**CG**	56	0.625	85	0.795
**CU**	58	1.274	69	1.377
**GA**	79	1.141	67	1.191
**GC**	107	1.194	114	1.067
**GG**	81	0.805	94	0.882
**GU**	44	0.861	45	0.901
**UA**	14	0.398	10	0.379
**UC**	38	0.834	54	1.078
**UG**	84	1.643	74	1.482
**UU**	22	0.847	12	0.513

Supplementary Table 3. HSVgB, Human, and Mouse Codon Usage Comparison

Codon	Amino Acid	HSVgB codon usage Fraction	Human codon usage Fraction	Mouse codon usage fraction	Codon	Amino Acid	HSVgB codon usage Fraction	Human codon usage Fraction	Mouse codon usage fraction
**UUU**	**F**	0.37	0.46	0.45	**UAU**	**Y**	0	0.44	0.43
**UUC**	**F**	0.63	0.54	0.56	**UAC**	**Y**	1	0.56	0.57
**UUA**	**L**	0	0.08	0.07	**UAA**	**Stop**	0	0.3	0.28
**UUG**	**L**	0.1	0.13	0.13	**UAG**	**Stop**	0	0.24	0.23
**CUU**	**L**	0.02	0.13	0.13	**CAU**	**H**	0.05	0.42	0.41
**CUC**	**L**	0.2	0.2	0.2	**CAC**	**H**	0.95	0.58	0.59
**CUA**	**L**	0.03	0.07	0.08	**CAA**	**Q**	0.08	0.27	0.26
**CUG**	**L**	0.66	0.4	0.39	**CAG**	**Q**	0.92	0.73	0.74
**AUU**	**I**	0	0.36	0.34	**AAU**	**N**	0.05	0.47	0.43
**AUC**	**I**	0.89	0.47	0.5	**AAC**	**N**	0.95	0.53	0.57
**AUA**	**I**	0.11	0.17	0.16	**AAA**	**K**	0.17	0.43	0.39
**AUG**	**M**	1	1	1	**AAG**	**K**	0.83	0.57	0.61
**GUU**	**V**	0.04	0.18	0.17	**GAU**	**D**	0.08	0.46	0.45
**GUC**	**V**	0.38	0.24	0.25	**GAC**	**D**	0.92	0.54	0.55
**GUA**	**V**	0.04	0.12	0.12	**GAA**	**E**	0.08	0.42	0.41
**GUG**	**V**	0.53	0.46	0.46	**GAG**	**E**	0.92	0.58	0.59
**UCU**	**S**	0.04	0.19	0.2	**UGU**	**C**	0.1	0.46	0.48
**UCC**	**S**	0.36	0.22	0.22	**UGC**	**C**	0.9	0.54	0.52
**UCA**	**S**	0	0.15	0.14	**UGA**	**Stop**	1	0.47	0.49
**UCG**	**S**	0.26	0.05	0.05	**UGG**	**W**	1	1	1
**CCU**	**P**	0.05	0.29	0.31	**CGU**	**R**	0	0.08	0.08
**CCC**	**P**	0.44	0.32	0.3	**CGC**	**R**	0.61	0.18	0.17
**CCA**	**P**	0.08	0.28	0.29	**CGA**	**R**	0.02	0.11	0.12
**CCG**	**P**	0.42	0.11	0.1	**CGG**	**R**	0.35	0.2	0.19
**ACU**	**T**	0.03	0.25	0.25	**AGU**	**S**	0.02	0.15	0.15
**ACC**	**T**	0.65	0.36	0.35	**AGC**	**S**	0.32	0.24	0.24
**ACA**	**T**	0	0.28	0.29	**AGA**	**R**	0	0.21	0.22
**ACG**	**T**	0.32	0.11	0.1	**AGG**	**R**	0.03	0.21	0.22
**GCU**	**A**	0.01	0.27	0.29	**GGU**	**G**	0.06	0.16	0.18
**GCC**	**A**	0.59	0.4	0.38	**GGC**	**G**	0.59	0.34	0.33
**GCA**	**A**	0.01	0.23	0.23	**GGA**	**G**	0.02	0.25	0.26
**GCG**	**A**	0.39	0.11	0.09	**GGG**	**G**	0.33	0.25	0.23

Supplementary Figure 1: Gating strategy for overall B cell immunophenotyping from splenocytes.

A) Representative flow cytometry gating strategy for overall B cell immunophenotyping. **B)** Percentages of transitional type 1 (T1) B cells, **C)** transitional type 2 (T2) B cells, and **D)** mature B cells were measured from splenocytes collected at week 10 from mice immunized with PBS, sGn-H (human) mRNA-LNP, or sGn-H (HSVgB) mRNA-LNP. Statistical analysis was determined by one-way ANOVA with Šídák's post hoc multiple comparison test. $p \leq 0.05$ is denoted by *, and *ns* indicates no significant difference.

Supplementary Figure 2: Overview of ELISpot images for detecting sGn-H-specific antibody-secreting cells (ASCs) in BALB/c mice.

A) Bone marrow from BALB/c mice (n=5 per group) immunized with PBS, sGn-H (human) mRNA-LNP, or sGn-H (HSVgB) mRNA-LNP were harvested at week 10. ELISpot assays were performed to detect sGn-H-specific ASCs. Bone marrow cells were serially diluted by a factor of two from 2.1×10^6 cells and seeded into Rows A-D or Row E-H. Each sample was seeded in duplicate. The rest of the wells were filled with serum-free RPMI1640 media only. For analysis, Rows C to D and G to H were selected to count sGn-H-specific ASCs per 1×10^6 cells. One sGn-H (HSVgB) mRNA-LNP-immunized mouse from Plate 2 columns 1 to 2 was removed from analysis for being an outlier in other experiments. The plate layouts were created with BioRender.com.

Supplementary Figure 3: Gating strategy for multiplex T cell activation-induced marker (AIM) assay.

A) Representative gating strategy used for identifying sGn-H-specific $CD4^+$ and $CD8^+$ T cells in the multiplex T cell AIM assay.

Supplementary Figure 4: sGn-H (HSVgB) mRNA-LNPs induce higher cytokine and chemokine production in BALB/c mice.

Splenocytes from BALB/c mice (*n*=5 per group) immunized with PBS, sGn-H (human) mRNA-LNP, or sGn-H (HSVgB) mRNA-LNP were harvested at week 10. These splenocytes were stimulated with sGn-H overlapping peptide pools, and the supernatants were collected for analysis. One mouse from sGn-H (HSVgB) mRNA-LNP group was excluded as an outlier. The remaining data from the Target 48 Mouse Cytokine panel are shown in **A)** other interleukin (IL) cytokine families, **B)** immune modulators and regulatory cytokines, and **C)** chemokines. Statistical analysis was performed by one-way ANOVA with Šídák's post hoc multiple comparison test. Red asterisks indicate significance between PBS and sGn-H (human) mRNA-LNP; blue asterisks between PBS and sGn-H (HSVgB) mRNA-LNP; black asterisks between sGn-H (human) mRNA-LNP and sGn-H (HSVgB) mRNA-LNP. $p \leq 0.05$ is denoted by *, $p \leq 0.01$ by **, $p \leq 0.001$ by ***, $p \leq 0.0001$ by ****, and *ns* indicates no significant difference.

Supplementary Figure 5: Comparison of mRNA stability of sGn-H (human) and sGn-H (HSVgB) in HEK293T cells.

A) Time course of sGn-H mRNA levels in HEK293T cells transfected with either sGn-H (human) mRNAs or sGn-H (HSVgB) mRNAs. Total RNA was collected at 6, 12, 24, 36, and 48 hours post-transfection (hpt). Ct values were normalized to human 18S rRNA, and

fold change was calculated relative to the 6 hpt sample. For plotting, the 6 hpt time point was set as 0 hour on the x-axis; thus 12, 24, 36, and 48 hpt correspond to 6, 18, 32, and 42 hours, respectively. Statistical analysis was performed using Student's t-test to compare relative sGn-H mRNA expression at each time point. *ns* indicates no significant difference.
